# Variability in P1 gene redefines phylogenetic relationships among cassava brown streak viruses

**DOI:** 10.1186/s12985-017-0790-9

**Published:** 2017-06-20

**Authors:** Willard Mbewe, Fred Tairo, Peter Sseruwagi, Joseph Ndunguru, Siobain Duffy, Ssetumba Mukasa, Ibrahim Benesi, Samar Sheat, Marianne Koerbler, Stephan Winter

**Affiliations:** 1 Department of Ecology, Evolution and Natural Resources, Rutgers University, New Brunswick, NJ 08901 USA; 2 School of Agriculture and Environmental Science, Department of Agricultural Production, Makerere University, P. O. Box, 7062 Kampala, Uganda; 3 Mikocheni Agricultural Research Institute, P. O. Box 6226, Dar es Salaam, PO Tanzania; 4 Chitedze Agricultural Research Station, P. O. Box, 153 Lilongwe, Malawi; 5 Leibniz Institute - DSMZ Plant Virus Department, Braunschweig, Germany

**Keywords:** Cassava brown streak viruses, Evolution, Diversity

## Abstract

**Background:**

Cassava brown streak disease is emerging as the most important viral disease of cassava in Africa, and is consequently a threat to food security. Two distinct species of the genus *Ipomovirus* (family *Potyviridae*) cause the disease: *Cassava brown streak virus* (CBSV) and *Ugandan cassava brown streak virus* (UCBSV). To understand the evolutionary relationships among the viruses, 64 nucleotide sequences from the variable P1 gene from major cassava producing areas of east and central-southern Africa were determined.

**Methods:**

We sequenced an amplicon of the P1 region of 31 isolates from Malawi and Tanzania. In addition to these, 33 previously reported sequences of virus isolates from Uganda, Kenya, Tanzania, Malawi and Mozambique were added to the analysis.

**Results:**

Phylogenetic analyses revealed three major P1 clades of Cassava brown streak viruses (CBSVs): in addition to a clade of most CBSV and a clade containing all UCBSV, a novel, intermediate clade of CBSV isolates which has been tentatively called CBSV-Tanzania (CBSV-TZ). Virus isolates of the distinctive CBSV-TZ had nucleotide identities as low as 63.2 and 63.7% with other members of CBSV and UCBSV respectively.

**Conclusions:**

Grouping of P1 gene sequences indicated for distinct sub-populations of CBSV, but not UCBSV. Representatives of all three clades were found in both Tanzania and Malawi.

## Background

Cassava (*Manihot esculenta* Crantz, Family: *Euphorbiaceae*) is an important staple food crop for over 800 million people across the globe [1]. Although cassava is known to be vulnerable to at least 20 different viruses, the two most economically damaging viral diseases in Africa are cassava mosaic disease and cassava brown streak disease (CBSD). The diseases have been associated with production losses worth more than US$1 billion every year [2]. Recent developments in cassava research have shown that CBSD is emerging as the most important viral disease of cassava in Africa, and is consequently a threat to food security [1]. Two distinct species of the genus *Ipomovirus* (family *Potyviridae*), *Cassava brown streak virus* [3] and *Ugandan cassava brown streak virus* (UCBSV [4, 5]) cause the disease. In this paper, both viruses are collectively called CBSVs. The characteristic symptoms of CBSVs include typical ‘feathery’ chlorosis and yellow patch symptoms along secondary and tertiary veins of older leaves of cassava, brown streaks on the stems, constriction in storage roots, and brown spots in the tuber visible when it is cut [6, 7]. Previously, CBSD was reported only from the coastal lowlands of East Africa, but recently it has spread throughout the Great Lakes region of East and Central-Southern Africa [8–14].

*Potyviridae* is a family of plant viruses with a single stranded, positive sense RNA genome and flexious, filamentous particles [10]. The monopartite +ssRNA genomes of the members of *Potyviridae* share similar genomic organization, with levels of amino acid identity in their polyproteins ranging from 42 to 56% among different species of the same genus and from 25 to 33% among viruses from different genera [11]. However, conservation of individual mature proteins varies. P1, a serine protease that self-cleaves at its C terminus and acts as an accessory factor for genome amplification (reviewed in [12] is the first protein of the polyprotein and the most variable in length and amino acid sequence [11]. Other roles of P1 include boosting the activity of the helper component protease (HCPro) to suppress RNA silencing [13] and to enhance the pathogenicity of heterologous plant viruses during coinfection [14, 15]. The genomes of CBSVs lack HCPro and the short P1 gene has RNA silencing suppression function [16]. Significantly divergent P1 gene sequences of CBSV have been found and recent studies have suggested that the P1 gene of CBSV (together with NIa, 6 K2, and NIb) have evolved more rapidly compared to other genes [17].

Genetic variability is an intrinsic feature of RNA viruses because of high mutation rates resulting from the lack of proofreading activity of their RNA-dependent RNA polymerases [14, 18]. RNA recombination events can additionally shape the diversity of populations of RNA viruses [15] which can lead to new phenotypes such as host range expansion [19]. Diversity among CBSV isolates was initially assessed using sequences at the conserved 3′-terminus of the RNA genome comprising the coat protein gene and parts of NIb [16] while comparative studies with complete viral genomes [5, 9, 20], have revealed more pronounced and distinctive features among virus isolates. In one previous study [5], sequence analysis of 7 virus isolates revealed two distinct CBSV sequence clades that were separated to the species level. Different biological features of members of these two clades provided justification for CBSVs to be assigned to two species: UCBSV and CBSV [5]. In that same study an isolate from coastal Tanzania (CBSV-Tan70, FN434473) was identified which was very similar to CBSV isolates throughout much of its genome, but with a strikingly different P1 gene which was equidistantly related to both CBSV and UCBSV isolates. As this divergent P1 region was found only in one CBSV isolate which otherwise had similar biological features than other CBSVs, further species delineation was not possible because of lack of similar isolates. The recent analyses of additional CBSV genome sequences from Tanzania [9] and from Uganda [16] revealed further diversity and also indicate the potential for an additional species or subspecies within the CBSVs.

In the study presented here, a total of 64 P1 gene sequences of CBSV isolates from major cassava producing areas of east and central-southern Africa were analysed. We sequenced a portion of the P1 gene from 31 isolates (from Malawi and Tanzania) and analyzed them with those previously reported from Uganda, Kenya, Tanzania, Malawi and Mozambique and present substantial evidence for the widespread occurrence of a distinct Cassava brown streak virus clade tentatively named CBSV-Tanzania (CBSV-TZ).

## Methods

### Source of virus isolates, amplification and sequencing

Cassava cuttings collected from CBSD-symptomatic plants in Malawi and Tanzania (Table 1) during national surveys in 2013 (under the auspices of each country’s agricultural research institutes). The plants were classified by having symptoms that were consistent with CBSD (feathery chlorosis along veins in leaves and brown streaks/lesions along the plant stem), or potentially were coinfected with agents causing both CBSD and CMD (mosaic, mottling, misshapen and twisted leaflets) and were taken to The Leibniz Institute – Deutsche Sammlung von Mikroorganismen and Zellkulturen GmbH (DSMZ) Plant Virus Department, where they were maintained under greenhouse conditions. Total RNA was extracted from the virus-infected leaves of the cassava plants using the cetyl trimethyl ammonium bromide method [21] with modifications described previously [22] or using an RNeasy Mini kit (Qiagen). Nucleic acids were quantified using a Nanodrop spectrophotometer, and about 2.0 × 10^−5^ μg/mL nucleic acid was used for virus detection by RT-PCR as detailed in Winter et al. [5]. A cDNA fragment, the partial sequence of the P1 gene, was amplified using virus specific primer sets designed by Winter et al. [5]. The reactions were performed in a GeneAmp 9700 PCR thermal cycler (Applied Biosystems, Foster City, CA, USA) set with the following conditions: 42 °C for 30 min for reverse transcription, followed by heat denaturation at 94 °C for 5 min; and then 35 cycles of amplification comprising the following: denaturation at 94 °C for 1 min, annealing at 52 °C for 1 min, extension at 72 °C for 1 min, followed by a single cycle of final extension at 72 °C for 10 min. All RT-PCR products were purified using a Qiagen gel extraction kit, ligated into the pDrive U/A cloning vector (Qiagen) and subsequently electroporated into *Escherichia coli* DH5α cells. The clones were Sanger sequenced in both orientations. A single consensus sequence for each isolate was verified to be CBSV by blastn searches of GenBank (https://blast.ncbi.nlm.nih.gov/Blast.cgi). The resulting nucleotide sequences were submitted to GenBank (pending accession numbers, Table 1).

**Table 1:** Cassava brown streak isolates used in this study

Isolate name	Location	Symptoms	Year collected	Country	Accession number	Reference
TZ_Chamb_1_13	Chambezi	CBSD-like	2013	Tanzania	KY290016	This study
TZ_Chamb_2_13	Chambezi	CBSD-like	2013	Tanzania	KY290017	This study
TZ_Chamb_3_13	Chambezi	CBSD-like	2013	Tanzania	KY290018	This study
TZ_Chamb_4_13	Chambezi	CBSD-like	2013	Tanzania	KY290019	This study
TZ_Chamb_5_13	Chambezi	CBSD-like	2013	Tanzania	KY290010	This study
TZ_Chamb_6_13	Chambezi	CBSD-like	2013	Tanzania	KY290011	This study
TZ_Chamb_7_13	Chambezi	CBSD-like	2013	Tanzania	KY290020	This study
TZ_Chamb_8_13	Chambezi	CBSD-like	2013	Tanzania	KY290012	This study
TZ_Chamb_9_13	Chambezi	CBSD-like	2013	Tanzania	KY290013	This study
TZ_Chamb_10_13	Chambezi	CBSD-like	2013	Tanzania	KY290014	This study
TZ_Chamb_11_13	Chambezi	CBSD-like	2013	Tanzania	KY290015	This study
TZ_Chamb_12_13	Chambezi	CBSD-like	2013	Tanzania	KY290021	This study
TZ_Chamb_13_13	Chambezi	CBSD-like	2013	Tanzania	KY290022	This study
TZ_Chamb_14_13	Chambezi	CBSD-like	2013	Tanzania	KY290023	This study
TZ_Unguja_1_13	Unguja	CBSD-like	2013	Tanzania	KY290024	This study
TZ_Mari_1_13	MARI	CBSD-like	2013	Tanzania	KY290025	This study
MW07	Karonga	CBSD-like	2013	Malawi	KY290003	This study
MW08	Karonga	CBSD-like	2013	Malawi	KY290004	This study
MW12	Karonga	CBSD-like	2013	Malawi	KY289996	This study
MW13	Karonga	CBSD-like	2013	Malawi	KY290006	This study
MW14	Rumphi	CBSD-like	2013	Malawi	KY289999	This study
MW15	Karonga	CBSD-like	2013	Malawi	KY289998	This study
MW16	NkhataBay	CBSD-like	2013	Malawi	KY289995	This study
MW22	Karonga	CBSD-like	2013	Malawi	KY290001	This study
MW23	Karonga	CBSD-like	2013	Malawi	KY290000	This study
MW37	NkhataBay	CBSD + CMD	2013	Malawi	KY290008	This study
MW40	Nkhotakota	CBSD-like	2013	Malawi	KY290002	This study
MW42	Karonga	CBSD-like	2013	Malawi	KY290005	This study
MW43	NkhataBay	CBSD-like	2013	Malawi	KY290007	This study
MW46	Lilongwe	CBSD + CMD	2013	Malawi	KY289997	This study
MW50	NkhataBay	CBSD-like	2013	Malawi	KY290009	This study
TZ-Nal:07	Naliendele	–	2007	Tanzania	HG965221	Bayene et al., unpublished
TZ:Kor6:08	Unknown	–	2008	Tanzania	GU563327	Mbanzibwa et al., 2011 [33]
TanZ	Kiabakari, Musoma	–	2008	Tanzania	GQ329864	Monger et al., 2010 [34]
Tan70	Mlandizi	–	2009	Tanzania	FN434437	Winter et al., 2010 [5]
MLB3	Muleba	–	2008	Tanzania	FJ039520	Mbanziwa et al., 2011 [33]
CBSV-Ug	Unknown	–	2006	Uganda	FJ185044	Monger et al., 2010 [34]
Mo_83	Namacurra	–	2009	Mozambique	FN434436	Winter et al., 2010 [5]
Ke_125	Kilifi	–	1999	Kenya	FN433930	Winter et al., 2010 [5]
Ke_54	Kilifi	–	1999	Kenya	FN433931	Winter et al., 2010 [5]
UGKab07	Kabanyolo	–	2007	Uganda	HG965222	Bayene, et al., unpublished
Ma_42	Chitedze	–	2007	Malawi	FN433932	Winter et al., 2010 [5]
Ma_43	Salima	–	2007	Malawi	FN433933	Winter et al., 2010 [5]
TZ:Ser 5	Serengeti	–	2013	Tanzania	KR108838	Ndunguru et al., 2015 [9]
UCBSV_TZ:Ser 6	Serengeti	–	2013	Tanzania	KR108837	Ndunguru et al., 2015 [9]
CBSV_TZ:Ser 6	Serengeti	–	2013	Tanzania	KR108830	Ndunguru et al., 2015 [9]
TZ-Tan 19-1	Tanga	–	2013	Tanzania	KR108834	Ndunguru et al., 2015 [9]
TZ-Tan 19-2	Tanga	–	2013	Tanzania	KR108832	Ndunguru et al., 2015 [9]
TZ-Tan 23	Tanga	–	2013	Tanzania	KR108839	Ndunguru et al., 2015 [9]
TZ-Tan 26	Tanga	–	2013	Tanzania	KR108833	Ndunguru et al., 2015 [9]
TZ-Nya 36	Nyasa	–	2013	Tanzania	KR108831	Ndunguru et al., 2015 [9]
TZ-Nya 38	Nyasa	–	2013	Tanzania	KR108829	Ndunguru et al., 2015 [9]
TZ-Mf-49	Mafia	–	2013	Tanzania	KR108828	Ndunguru et al., 2015 [9]
TZ-Maf-51	Mafia	–	2013	Tanzania	KR108836	Ndunguru et al., 2015 [9]
TZ-Maf-58	Mafia	–	2013	Tanzania	KR108835	Ndunguru et al., 2015 [9]
F21S4_S11_N	Unknown	–	2013	Kenya	KR911736	Kathurima et al., 2016 [32]
F25S6_S10_N	Unknown	–	2013	Kenya	KR911725	Kathurima et al., 2016 [32]
F3S1_S23_C	Unknown	–	2013	Kenya	KR911727	Kathurima et al., 2016 [32]
F16S4_S7_W	Unknown	–	2013	Kenya	KR911723	Kathurima et al., 2016 [32]
F18S1_S3_W	Unknown	–	2013	Kenya	KR911726	Kathurima et al., 2016 [32]
F10S2_S20_C	Unknown	–	2013	Kenya	KR911722	Kathurima et al., 2016 [32]
F9S2_S21_C	Unknown	–	2013	Kenya	KR911721	Kathurima et al., 2016 [32]
F17S3_S2	Unknown	–	2013	Kenya	KR911724	Kathurima et al., 2016 [32]
F19SS2_S4_W	Unknown	–	2013	Kenya	KR911729	Kathurima et al.,2016 [32]

### Nucleotide similarity and putative recombination breakpoint analysis

Percentage nucleotide identities were computed in Geneious Software v10.0.5 [23]. A matrix of nucleotide identities was produced using the Sequence Demarcation Tool v1 [24]. Putative recombination events were detected using nine recombination detection programs within the RDP4 package (http://darwin.uvigo.es/rdp/rdp.html): RDP, GENECONV, MaxChi, Chimaera, Bootscan, Siscan, PhylPor, LARD, and 3Seq [25]. Analyses were carried out using default settings (except sequences were set to linear) and the Bonferroni correction *P*-value cut-off of 0.05. Only breakpoints supported by at least three methods were considered further [26].

### Phylogenetic analysis

Phylogenetic relationship among P1 regions of CBSV isolates (Table 1) was determined. The sequences were aligned using ClustalW [27] in MEGA 7 [28] and edited manually. The alignment was trimmed to give all sequences uniform length. MEGA 7 was used to construct maximum likelihood (ML) phylogenetic trees, and editing was done in FigTree v1.4.2 (http://tree.bio.ed.ac.uk/software/figtree/). The trees were created using a GTR nucleotide substitution model, and the best tree was bootstrapped with 1000 replicates [29].

## Results

To examine the genetic diversity of CBSVs, field surveys and extensive sampling were performed in Malawi and Tanzania in 2013, yielding a total of 31 newly sequenced isolates (16 from Tanzania and 15 from Malawi). Thirty-three other previously published P1 sequences of CBSVs were retrieved from GenBank and aligned with these new sequences and a sister taxon, *Sweet potato mild mottle virus* [3]. The alignment (510 nt) was found to be free of detectable recombination.

**Fig. 1:**
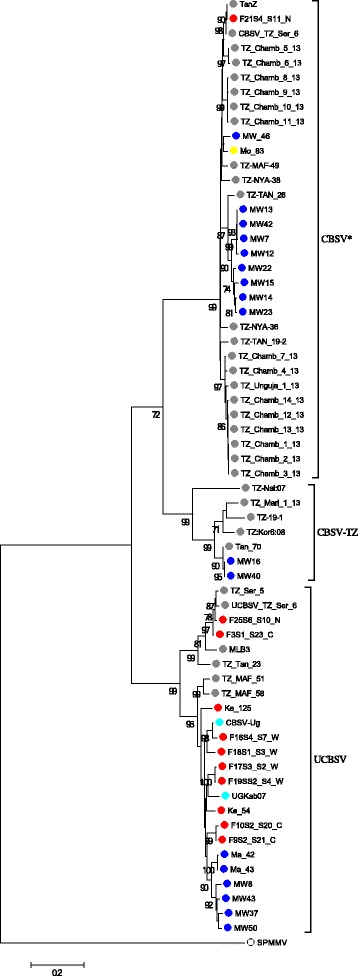
ML tree of partial P1 gene sequences of Cassava brown streak viral isolates (Table 1). Sequences are from Tanzania (*green*), Mozambique (*yellow*), Kenya (*red*), Uganda (*purple*) and Malawi (*blue*). Bootstrap values higher than 70% are shown. The scale is in substitutions/site

**Fig. 2:**
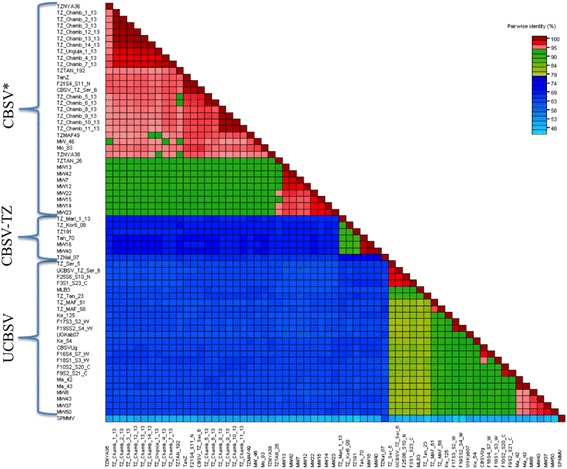
Pairwise identity matrix generated from CBSV partial P1 gene sequences. Each colored key represents a percentage to the identity score between two sequences

A phylogenetic tree generated from these 64 partial P1 sequences confirmed significant genetic variability among CBSVs and unambiguously resolved three clades. Seven isolates; five from Tanzania (TZ-Nal:07, TZ_Mari_1_13, TZ:Kor6:08, TZ-19-1, Tan_70) and two from Malawi (MW16, MW40) formed a clade which is significantly divergent from other CBSV isolates (we term this clade CBSV*) and the UCBSV isolates respectively (Fig. 1). We have tentatively named this group CBSV-Tanzania as it is more closely related to CBSV than UCBSV isolates and contains sequences predominantly from Tanzania. The clade includes the CBSV isolate Tan_70 from coastal Tanzania which was previously reported [5]. Isolates belonging to the CBSV-TZ clade were closely related, sharing P1 gene sequences very different from those in the CBSV* and UCBSV clades (Fig. 2). P1 sequences in the CBSV-TZ clade have low sequence identity with P1 gene sequences of isolates in the CBSV* (63.2 to 70.9%) and UCBSV (62.0 to 65.4%) clades.

## Discussion

As CBSD continues to threaten subsistence cassava production in east, central and southern Africa, there is a need to understand dynamics of viral diversity as this has implications on evolution and emergence of new species or strains. This is especially critical in light of the rapid spread of the disease from the Great Lakes region of east and central-southern Africa [8, 9, 11–13, 20]. We present here an analysis of 64 partial P1 sequences of cassava brown streak viruses from cassava growing regions of Africa where CBSVs are known to occur. Considerable variance of gene size and sequence within P1 genes of the family *Potyviridae* has been previously reported [17, 14] indicating that P1 is an ideal region to reveal population differentiation and incipient speciation within cassava ipomoviruses. Further, whole genome analyses of CBSVs had previously identified unusual sequence diversity in P1 [5]. Our phylogenetic analysis revealed that the CBSVs sequences formed three distinct clades (Figure 1). In addition to the previously characterized species UCBSV and CBSV, the novel clade which includes the Tan_70 isolate [5] presents a major sub-group of CBSV, for which we propose the tentative name CBSV-Tanzania.

Another study on variation of CBSVs, based on short coat protein fragments (~230 nt) revealed a number of viruses that are intermediate between the two CBSV and UCBSV species subgrouping, and consequently presented a hypothetical possibility of a new novel species or sub-species associated with CBSVs [30]. Recent whole genome analyses of UCBSV isolates [9] suggested further speciation among isolates of UCBSV from Tanzania. Our results, concentrating on the analysis of the variable P1 gene and additional virus isolates from east and central-southern Africa, confirm the diversity observed with the in other studies and provides evidence from P1 gene analysis for subdivision of CBSV and the presence of the clade CBSV-TZ. Our results also show that the Malawi and Tanzania viruses are more diverse than those found in Kenya, Uganda, and Mozambique (Figure 1). That Tanzania has qualitatively higher diversity of CBSVs may not just be due to increased surveillance and sampling there; while UCBSV is distributed all over Malawi, CBSV* and sub-group CBSV-TZ are localized in northern Malawi, bordering Tanzania [30]. Movement of cultivars between the two countries could help to explain the shared diversity of CBSVs, which could be due to either purely geographical reasons or unique adaptations of circulating CBSV-TZ to locally popular cassava cultivars.

While the region around Lake Malawi was where CBSD was first observed [6] the higher prevalence and wide distribution of UCBSV compared to CBSV throughout Malawi, Tanzania and surrounding countries suggest that UCBSV was likely the virus implicated in the first finding of CBSD. Comparisons of full genome sequences of Malawian CBSVs with those of CBSVs obtained from CBSD-affected areas of neighboring countries (Tanzania and Mozambique) would likely clarify questions about the evolutionary history and biogeography of the viruses in the region. Regardless, it is clear that the CBSVs do not have geographically distinct distributions as was previously hypothesized [4].

Studies by Ndunguru et al. [9] showed that a previously described CBSV Tanzanian isolate TZ-Nal 07 had a recombination event in the 5′ end in the P1 gene. The P1 region is known to harbor obvious recombination in several potyviruses [31] and contributes to its overall variability. Although our final dataset did not statistically support recombination breakpoint (s) within P1, when diverse isolates from Kenya [32] were left out of the analysis, the isolate (TZ-Nal 07) was identified by two methods as a putative recombinant between a member of the CBSV-TZ clade (TZ:Kor6:08) and CBSVMo_83 (data not shown). This recombination event may be better supported from the full genome dataset [9] but the finding is consistent with the phylogenetic placement of TZ-Nal 07 as basal to the CBSV-TZ clade. However, we have no evidence for recombination being the origin for this well-supported subgroup and hence the diversification of P1 in the genomes of CBSV-TZ isolates still requires further investigation.

## Conclusions

Our in-depth look at CBSVs from Malawi and Tanzania has revealed that the divergent Tan_70 isolate is in good company, and that the CBSVs have three separable groups of diverse P1 gene sequences. Further research will establish if the variable P1 region is an accurate bellwether for overall population divergence, and future phenotypic characterization will determine whether CBSV-TZ represents a novel strain or subspecies of CBSV.

## Abbreviations

CBSD: Cassava brown streak disease
CBSV: Cassava brown streak virus
CBSV-TZ: Cassava brown streak Tanzania
cDNA: complementary Deoxyribonucleic Nucleic Acid
CMD: Cassava mosaic disease
MEGA: Molecular Evolutionary Genetics Analysis
ML: Maximum Likelihood
RNA: Ribonucleic Acid
RT-PCR: Reverse Transcriptase – Polymerase Chain Reaction
SDT: Sequence Demarcation Tool
UCBSV: Ugandan cassava brown streak virus
